# A Novel DBL-Domain of the *P. falciparum* 332 Molecule Possibly Involved in Erythrocyte Adhesion

**DOI:** 10.1371/journal.pone.0000477

**Published:** 2007-05-30

**Authors:** Kirsten Moll, Arnaud Chêne, Ulf Ribacke, Osamu Kaneko, Sandra Nilsson, Gerhard Winter, Malin Haeggström, Weiqing Pan, Klavs Berzins, Mats Wahlgren, Qijun Chen

**Affiliations:** 1 Department of Parasitology, Mycology and Environmental Microbiology (PMV), Swedish Institute for Infectious Disease Control (SMI), Karolinska Institutet, Stockholm, Sweden; 2 Department of Microbiology, Tumour and Cell Biology (MTC), Karolinska Institutet, Stockholm, Sweden; 3 Center for Infectious Medicine (CIM), Karolinska Institutet, Stockholm, Sweden; 4 Department of Molecular Parasitology, Ehime University Graduate School of Medicine, Ehime, Japan; 5 Department of Etiologic Biology, Second Military Medical University, Shanghai, China; 6 Department of Immunology, Wenner-Gren Institute, Stockholm University, Stockholm, Sweden; Federal University of São Paulo, Brazil

## Abstract

*Plasmodium falciparum* malaria is brought about by the asexual stages of the parasite residing in human red blood cells (RBC). Contact between the erythrocyte surface and the merozoite is the first step for successful invasion and proliferation of the parasite. A number of different pathways utilised by the parasite to adhere and invade the host RBC have been characterized, but the complete biology of this process remains elusive. We here report the identification of an open reading frame (ORF) representing a hitherto unknown second exon of the Pf332 gene that encodes a cysteine-rich polypeptide with a high degree of similarity to the Duffy-binding-like (DBL) domain of the erythrocyte-binding-ligand (EBL) family. The sequence of this DBL-domain is conserved and expressed in all parasite clones/strains investigated. In addition, the expression level of Pf332 correlates with proliferation efficiency of the parasites *in vitro*. Antibodies raised against the DBL-domain are able to reduce the invasion efficiency of different parasite clones/strains. Analysis of the DBL-domain revealed its ability to bind to uninfected human RBC, and moreover demonstrated association with the iRBC surface. Thus, Pf332 is a molecule with a potential role to support merozoite invasion. Due to the high level of conservation in sequence, the novel DBL-domain of Pf332 is of possible importance for development of novel anti-malaria drugs and vaccines.

## Introduction

The invasion of RBC by a *Plasmodium*-merozoite is a cascade like process involving adhesion, reorientation, junction-formation and invagination. This procedure requires close interaction between the parasite derived ligands and the host receptors on the RBC surface (reviewed by Gaur et al. [1]). Interestingly, a majority of *Plasmodium falciparum* merozoites released from ruptured schizonts fail to invade new RBC *in vitro*. Further, *P. falciparum* purified merozoites do not consistently infect RBC [2], [3] while in contrast isolated merozoites of both murine and primate malaria parasites easily invade and proliferate within new RBC [3]. It has therefore been speculated that parasite derived molecules adhering to the merozoites are essential elements facilitating the invasion process [4] or that a specific interaction between infected and uninfected RBC prior to invasion may be necessary [5].

*P. falciparum* displays numerous parasite derived proteins on the merozoite surface that fulfil important functions for the multiplication of the parasite; the most important ones characterized so far are the Merozoite surface protein 1 (MSP-1) and the Apical membrane protein 1 (AMA-1), which are essential for the parasites survival [6], [7]. During the invasion process, the parasite forms a tight junction with the RBC membrane involving several proteins discharged from micronemes and rhoptries in the apical part of the cell. Among these are the erythrocyte binding proteins of the EBL-family located in the micronemes and the reticulocyte binding like (RBL) proteins situated at the neck of the rhoptries, which are expressed simultaneously by the merozoite. However, it is probably the available receptor on the RBC surface that determines which ligand the parasite employs for invasion [8].

EBA-175 was the first protein identified to be involved in the junction formation between merozoite and RBC mediating binding to the receptor glycophorin A (reviewed by Gaur et al. [1]), followed by EBA-175 paralogues such as EBA-140 and EBA-181 binding to glycophorin C or unknown receptors [9]. All members of the EBA-family are featured by an N-terminal signal sequence followed by a cysteine-rich motif with one or two Duffy-binding like domains, a C-terminal cysteine-rich motif, a transmembrane (TM) domain and a short cytoplasmic tail. Parasite mutants deficient in single members of the EBA-family do not show a significantly reduced merozoite invasion rate, as demonstrated by BAEBL/EBA-140 or JESEBL/EBA-180 knockout clones that maintain a comparable RBC invasion rate as wild type parasites [10], [11]. However, single amino acid alterations in the EBA-140 or EBA-181 sequences will lead to a change in receptor specificity, while sequence polymorphism in EBA-175 does not change its receptor-binding specificity [10].

A recent study by Glushakova [12] illustrates that merozoites invade RBC more efficiently when the iRBC are bound to uninfected RBC prior to schizont rupture and merozoite release. Molecules that participate in this interaction between iRBC and RBC may likewise play an important role in the initial adhesion process between merozoite and RBC [9]. A candidate molecule mediating the interaction between late stage iRBC and RBC is the polypeptide Pf332 (Antigen 332), which was first identified by Mercereau-Puijalon [13], [14]. Pf332 is highly glutamic acid rich and contains long repetitive sequences in an internal region named EB200, and a conserved tryptophan-rich domain (WRD) with similarities to that of PfEMP-1, SURFIN, and PkSICAvar [4]. Antibodies targeting this region can block the adhesion of iRBC to cells expressing CD36 and efficiently inhibit parasite invasion [15]–[17]. However, antibodies to the glutamic-acid reach repeats cross-react with several other *P. falciparum* proteins including Pf11-1, RESA/Pf155 with similar repetitive sequences, and the specificity of its inhibitory effect has therefore not been conclusive. Recent studies suggest that Pf332 assembles together with a number of different surface-related proteins such as the RIFINs and PfEMP1 in the erythrocyte-cytoplasm to be directed together to the erythrocyte membrane [18] although the biological function of Pf332 has remained unclear.

Here we propose that the open reading frame of Pf332 as originally described [13], [19] is in fact only the second exon of a giant gene comprising two exons. We identified a previously unrecognized Pf332 exon I, which is present in all parasite clones/strains investigated and encodes an erythrocyte-binding DBL-domain together with a transmembrane domain. Characterization of both the native and *in vitro* expressed Pf332 DBL-domain revealed its surface association and the ability to bind to human RBC. Significantly, antibodies raised against this region were able to reduce the invasion efficiency *in vitro* independent of geographical origin or receptor specificity of the *P. falciparum* clone/strain. This suggests the conserved polypeptide encoded by exon I of Pf332 exhibit a functional role during the RBC invasion process and is therefore a potential novel candidate for a blood stage vaccine.

## Results

### The gene encoding Pf332 consists of two exons separated by a short intron

During a survey to identify open reading frames (ORF) containing domains able to bind to RBC, the ORF PF11_0506 was identified, for which the adjacent ORF PF11_0507 encoding Pf332 started only 280 bp after the predicted stop codon of PF11_0506. The length of the intergenic region is very short compared to the mean length in *P. falciparum* (1694 bp) [20] and was thus assumed to be an intron rather than an intergenic region.

**Figure 1:**
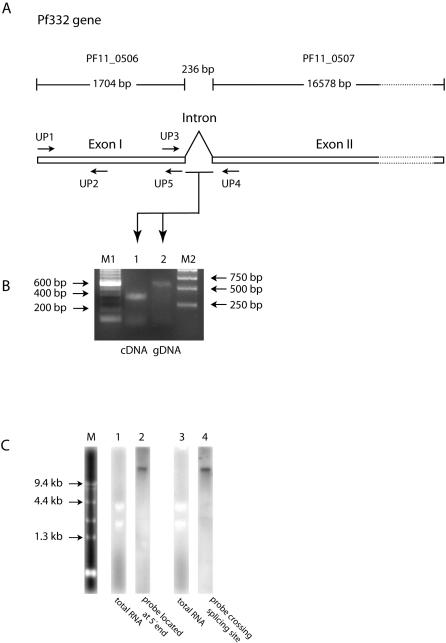
Structural analysis of the gene encoding Pf332. A. Schematic structure of the Pf332 gene. The gene is composed of a 5′ exon I (PF11_0506) with a size of 1704 bp and a 3′ exon II (PF11_0507), separated by a short intron of 236 bp. The 3′-exon II is the gene fragment previously referred to as the gene coding for the antigen Pf332. The location of the primers is indicated as arrows. B. PCR amplification from cDNA and gDNA across the intron region of the Pf332 gene: Lane 1: PCR amplification with primers UP3 and UP4 from cDNA; lane 2: PCR amplification with the same primer pair from gDNA. C. Northernblot with probes located at 5′-end (UP1–UP2) and across the splicing site (UP3–UP4) of the Pf332 gene. Total RNA from 3D7AH1 iRBC was resolved in an agarose gel (lanes 1 and 3). Lane 2 shows the hybridisation with the first probe (UP1–UP2); lane 4 shows the hybridisation with the second probe (UP3–UP4) revealing a band of the same size as seen in the first hybridization.

PCR amplification from gDNA and cDNA of the 3D7AH1 parasite clone was performed using a set of primers, where the forward primer UP3 was located at the 3′end of PF11_0506 and the reverse primer UP4 at the 5′end of PF11_0507 (Pf332). The size of the RT-PCR products from cDNA was 350 bp (Fig. 1A), whereas the size of the PCR products was 600 bp if gDNA of the clones 3D7AH1 (Fig. 1B) or FCR3S1.2 (data not shown) were used as a template. Comparison of the sequences of the PCR and RT-PCR products revealed an intron sequence possessing typical ‘gt-ag’ splicing sites at its ends.

To further confirm that the two transcripts of PF11_0506 and PF11_0507 constitute a single mRNA, Northern blot hybridisations were performed with two different probes. Probe 1 was located at 5′-end of PF11_0506 (amplified with UP1 and UP2, Fig 1A), while probe 2 covered the splicing sites (amplified with UP3 and UP4, Fig 1A). RNA was purified from the 3D7AH1 clone at around 20 h post invasion (p.i.). The two probes hybridised to a single transcript of a size considerably higher than 9.4 kb (Fig. 1C).

Combining these data, it can be concluded that the two ORFs (PF11_0506 and PF11_0507) annotated in the 3D7 genome database, as distinct genes are actually two exons of a single gene encoding Pf332. The full size of the mRNA (3D7AH1) is 18282 bp encoding a polypeptide of 6094 amino acids with a predicted molecular weight of approximately 670 kDa.

### The sequence of exon I of Pf332 is conserved in *P. falciparum*

A single DNA fragment was amplified from 10 different *P. falciparum* clones/strains originally isolated from various geographical locations and one Ugandan patient isolate. PCR amplification was conducted with a pair of primers (UP1, UP5) specific to the sequence of the 3D7AH1 clone (Fig. 1A and Fig. S1). When digested with the restriction enzyme EcoR I (3 digestion sites are present in the 3D7AH1 sequence, data not shown) the PCR products did not show any restriction fragment length polymorphism (data not shown). In addition, the DNA fragments of 11 parasite clones/strains (3D7AH1, NF54, FCR3S1.2, TM284S2, Dd2, FCB-3, K1, R29, FCR3CSA, F32 and UAS22) encoded by exon I of the molecule were sequenced and only four point mutations were found (in K1, 3D7AH1 and Dd2; Table 1). In contrast, sequence polymorphism was mainly reported in the repetitive region of exon II [14], as often seen in molecules important during sporozoite and merozoite invasion such as the repetitive regions of the circumsporozoite protein (CSP) and the block two sequence of MSP-1.

**Table 1:** Amino acid substitutions in the region encoded by exon I of Pf332

Parasite clone/strain	Position 69	Position 117	Position 329	Position 363
Dd2	Asn	Thr	Asn	*Thr*
FCR3S1.2	Asn	Thr	Asn	Met
FCB3	Asn	Thr	Asn	Met
F32	Asn	Thr	Asn	Met
K1	*Cys*	Thr	*Asp*	Met
UAS22	Asn	Thr	Asn	Met
TM284S2	Asn	Thr	Asn	Met
3D7AH1	Asn	*Ala*	Asn	Met

The amino acid residues of the consensus sequence of exon I of Pf332 are shown for 8 different parasite strains/clones, substitutions found in these sequences are visualized in italics.

### Exon I of Pf332 encodes for a DBL-domain with high similarity to the DBL-domains of the EBL-protein family

**Figure 2:**
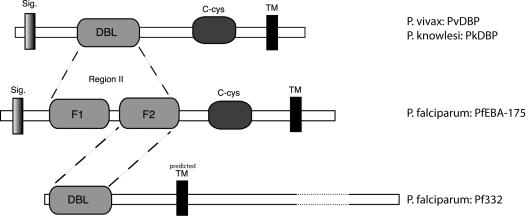
Schematic illustration of the conserved structure of the erythrocyte-binding region of Pf332 and the EBL family. The N-terminal region of the Pf332 encoded by exon I contains a Duffy-binding like domain homologous to the DBL-domain of the EBL-family. This domain of Pf332 is located closer to the N-terminal part of the molecule and lacks a signal leading sequence as compared to the other EBL family members. It aligns with the F2 region of EBA-175 of P. falciparum and the DBP of P. vivax. In addition, the carboxyl cysteine (c-cys)-rich domain present upstream of the transmembrane domain in EBA-175 and DBP is not found in Pf332.

BLASTP search and sequence alignment of the amino acid sequence encoded by the ORF PF11_0506 revealed that the N-terminal region (aa 1-250) possesses a domain homologous to the DBL-domains of the EBL-family including e.g. the EBA-175 of *P. falciparum* (Fig. S2) [21], although the overall protein structure of Pf332 differs from this protein family. Pf332 does neither contain a N-terminal segment (NTS) nor a signal leader sequence upstream of the DBL-domain (Fig. 2). The DBL-domains of the other EBL-family members are located further to the centre of the molecule, downstream of a NTS of variable size. Further, all EBL-family proteins presented an N-terminal signal peptide sequence.

Sequence comparison of the EBL-family proteins and the Pf332 showed that they share conserved cysteine residues and other amino acid residues, which are of importance for maintaining the structure of the molecule. Among 12 Cysteine-residues 5 are conserved indicating the formation of at least two disulfide bonds within the molecule (Fig. S2). The DBL-domain of Pf332 is more similar to the F2 than to the F1 region of the EBA-175 protein (Fig. 2); however, the absence of the WWXXXXXXXW sequence motif commonly found in other EBL family members suggests a distinct RBC-binding function for this domain.

Alignment with the EBL-family also reveals that Pf332 does not have a C-terminal Cysteine-rich domain after the DBL-domain. In contrast, Pf332 has a putative transmembrane region, located close to the DBL-domain (aa 540–560) (Fig. 2). Four membrane targeting motifs (RxLxE/Q) were found in the N-terminal region (aa 77–81), in the middle (aa 2628–2632) and close to the end (aa 4537–4541, 4922–4926) of the molecule, indicating that Pf332 is a transmembrane protein with a similar transport pathway as other molecules directed to the surface, such as PfEMP1 [22], [23].

### Pf332 is expressed by all parasites studied

**Figure 3:**
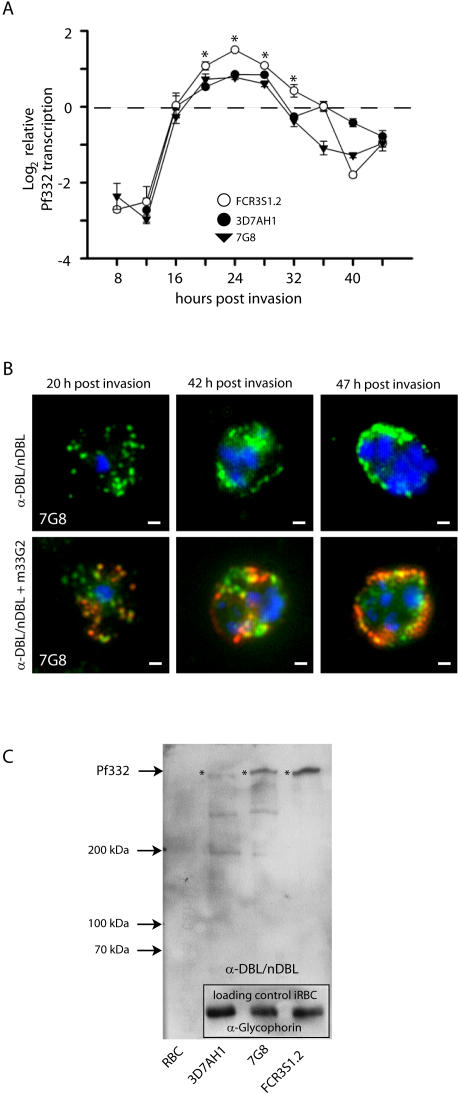
Transcription and expression of Pf332 in different P. falciparum clones/strains. A: The level of Pf332 mRNA transcription, relative to the endogenous control seryl-tRNA synthetase, was determined by real-time quantitative PCR. Normalized Pf332 levels at different times p.i. are plotted for 3D7AH1 (•), FCR3S1.2 (○) and 7G8 (▾). Error bars represent the standard deviation of the quotient and * denotes significantly higher levels of transcription in FCR3S1.2 (p<0.001, One Way ANOVA with Tukey test). The amount of Pf332 mRNA transcripts in FCR3S1.2 is around two fold higher than in 3D7AH1 and 7G8 at the time of maximal transcription. B: Upper panel: Immunofluorescence staining with α-DBL/nDBL-domain antibodies of Pf332. α-DBL/nDBL-domain antibodies (green) visualize at early trophzoite stage (around 20 h p.i.) vesicles that carry Pf332 in the cytosol of the iRBC; when the iRBC reaches late trophozoite/schizont stage (40–47 h p.i.), Pf332 can be observed in association with the iRBC membrane. Lower panel: Additional staining with the α-Pf332 monoclonal antibody m33G2. Staining with previously raised sera against the Pf332 (m33G2, red) show that α-DBL/nDBL-domain antibodies (green) co-localize with sera raised against the polypeptide encoded by exon II of Pf332. Staining is shown for the parasite strain 7G8, DNA staining with Hoechst (blue); Scale bars = 1 µm. C: Immunoblot analysis confirmed that antibodies raised against the DBL/nDBL-domain of Pf332 reacted with the same high molecular weight polypeptide as the previously raised antibodies α-EB200 and m33G2 (compare Fig. S3). This verifies that the sequence of the Pf332-DBL/nDBL-domain is an additional exon of the same open reading frame as the previously described Pf332. The total amount of expressed Pf332 varies in between parasites, with FCR3S1.2 iRBC expressing the highest amount of Pf332. An α-glycophorin antibody was used compare the amount of loaded material in the different lanes. The bands corresponding to Pf332 are marked with an asterix.

Microarray data illustrate that the transcription pattern of the Pf332 gene is similar in all investigated parasite clones/strains (HB3, 3D7, Dd2) [24], [25]. This was confirmed by real-time quantitative PCR analysis using RNA of three parasite clones/strains (3D7AH1, FCR3S1.2, 7G8) collected every 4 hours from 8 h p.i. onwards. The primers used were specific to the 5′ region of the transcript. Transcription was normalized to *seryl-tRNA synthetase* as an internal standard control. Results (Fig. 3A) clearly showed that the gene encoding Pf332 is activated in all three parasite clones/strains at approximately 16 h reaching maximal transcription at 24 h p.i.. Although all parasite clones/strains displayed the same transcription pattern, the level of transcription varied considerably among the different clones/strains, with FCR3S1.2 iRBC showing a transcription rate twice as high as the 3D7AH1 and 7G8 iRBC (Fig. 3A).

In addition, Pf332 expression was investigated in 3D7AH1, FCR3S1.2 and 7G8 iRBC by immunofluorescence and Western blot analysis with antibodies specific to the N-terminal region (α-Pf332-DBL/nDBL) as compared to antibodies towards the repetitive region (EB200) of the molecule and the human monoclonal antibody m33G2 [16] (Fig. 3B, C and Fig. S3). Expression of Pf332 starts immediately after initiation of transcription [18] and reaches maximal levels at 36 h p.i.. Immunofluorescence with α-Pf332-DBL/nDBL sera visualized that the protein is found within the parasitophorous vacuole at the ring stage, while with maturation of the parasite it is transported through the iRBC cytoplasm and directed towards the surface of the host cell, as previously observed with sera against other regions of the molecule (Fig. 3B).

Further, FCR3S1.2 iRBC displayed a higher expression rate of Pf332 as compared to the two other strains (Fig. 3C) corresponding to the transcription patterns visualized by quantitative PCR. Interestingly, the parasite clone FCR3S1.2 has the fastest multiplication rate among the three clones/strains investigated, indicating that Pf332 might be a protein that supports the parasite invasion process.

### The DBL-domain of Pf332 is surface associated and binds to human RBC *in vitro*

**Figure 4:**
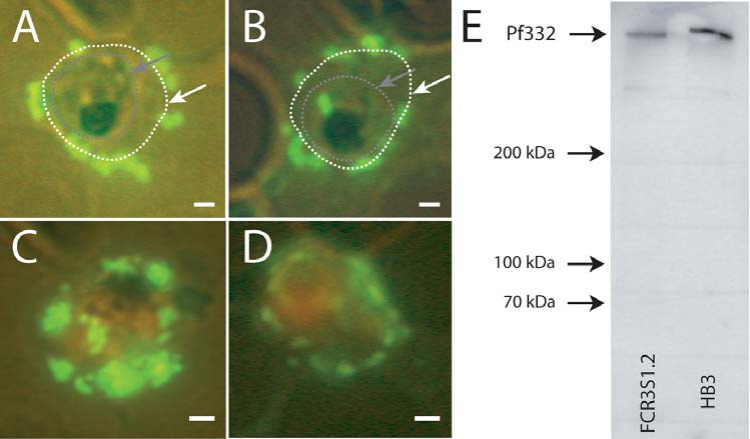
The Pf332 DBL-domain is iRBC surface associated. Immunofluorescence with antibodies against the DBL-domain visualizes the surface association of the Pf332 DBL-domain as shown for HB3 iRBC (A+C) and FCR3S1.2 iRBC (B+D). A+B: Parasites are unstained; the RBC membrane is indicated with a white line/arrow, the intracellular parasite with a grey line/arrow. C+D: Parasites are stained orange with Ethidium-bromide. Scale bars = 1 µm. E: Western blot with the same α-Pf332 DBL-domain antibody as used for the immunofluorescence assays; the antibody recognizes only the molecule Pf332 and does not cross-react with any other proteins.

Immunofluorescence assays on live trophozoite iRBC (HB3, FCR3S1.2) at 36 to 40 h p.i. using a serum raised against the DBL-domain of Pf332 visualized that this domain is associated with the surface of the iRBC (Fig. 4 A–D). In order to exclude that the observed reactivity results from uptake of the antibody into the iRBC, respectively leaky membranes as often observed in late stage iRBC, IFA assays were in addition carried out with various control proteins, which are parasite-derived, but not exposed on the iRBC surface. Antibodies against the ATS-domain of PfEMP1, PfEMP2 as well as an unrelated control protein did not show any reactivity in these assays.

**Figure 5:**
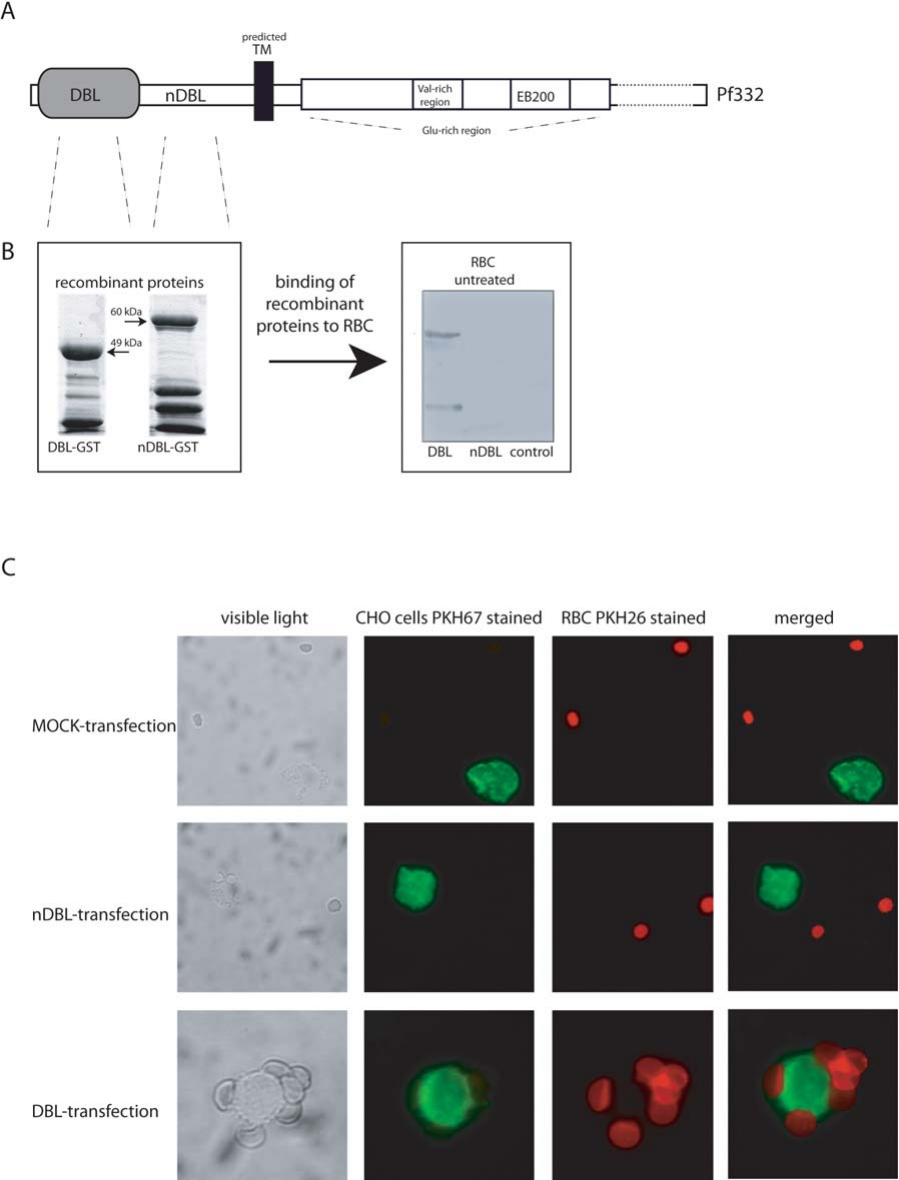
The DBL domain of Pf332 binds to human RBC. A: The protein Pf332 consists of a potentially extracellular region encoded by exon I and is composed of a DBL- and nDBL-domain followed by a predicted transmembrane domain. The region of the molecule originally described as Pf332 is encoded by a second exon and contains a Glutamic-acid rich part, which includes a Valin-rich area as well as the region EB200 build of numerous repeats of a XXXEEXXEEXX motif (X = hydrophobic aa). B: The Pf332-DBL- and Pf332nDBL-region were expressed as two individual GST-fusion proteins in E.coli. Binding assays, in which human RBC were incubated with the DBL-, nDBL- and an unrelated control protein were carried out. Binding of the proteins was visualized by subjecting the RBC to immunoblotting, where the bound protein was detected with an α-GST antibody. The DBL- but not the nDBL-domain or the unrelated control protein was able to bind to human RBC. C: Transient transfected CHO cells expressing either the DBL- or nDBL-domain on their surface were tested for their ability to bind to human RBC. CHO cells were stained red with PHK26 and incubated with RBC stained green with PKH67. CHO-cells expressing the DBL-domain avidly bound RBC, lower panel, while CHO cells expressing the nDBL-region or MOCK-transfected cells did not show any binding towards RBC (middle and upper panel).

In order to investigate the function of the surface associated DBL-domain, recombinant proteins (tagged with Gluthatione-S-Transferase, GST) corresponding to either the DBL-domain (Pf332-DBL) or nonDBL-domain (Pf332-nDBL) (Fig. 5A) of Pf332 were generated in *E. coli*. These recombinant domains were assayed for their capacity to bind to human RBC. The Pf332-DBL-domain bound to the surface of human RBC, while neither the recombinant Pf332-nDBL-domain or an unrelated GST-tagged control protein showed any affinity to human RBC (Fig 5B). In additional experiments, receptors on the RBC surface were modified with different enzymes. Treatment of RBC with neuraminidase did not abolish the binding of the recombinant Pf332-DBL-domain suggesting that the receptor of Pf332 is not a glycophorin molecule, likewise treatment of the RBC with trypsin or heparitinase did not influence the binding of the Pf332-DBL-domain (data not shown).

To further illustrate the binding capacity of the DBL-domain, CHO cells transiently expressing the DBL- or the nDBL-domain on their surface were constructed and tested for their ability to bind to human RBC. After transfection, 30–35% of the CHO cells were positive for expression of the different domains. The cells presenting the Pf332-DBL-domain were found to have RBC attached to their surface, while the presence of either the Pf332-nDBL-domain or MOCK transfection did not result in any RBC binding activity (Fig. 5C).

### Antibodies against Pf332 decrease parasite invasion efficiency

**Figure 6:**
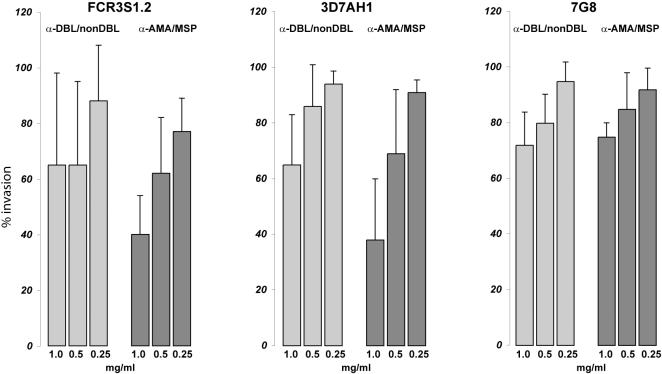
Antibodies directed against the DBL-domain of Pf332 inhibit parasite invasion. RBC infected with the parasite clones/strains FCR3S1.2, 3D7AH1 and 7G8 were cultivated in the presence of α-Pf332-DBL/nDBL-antibodies for 24 h allowing reinvasion of the parasites into new RBC. The parasitemia was analysed by flow-cytometry and compared to a control cultivated in the presence of a serum raised against a non-related protein. Parasites displayed a decreased invasion rate in the presence of α-Pf332-DBL/nDBL-antibodies. The effect of α-Pf332-DBL/nDBL-antibodies (light bars) is slightly lower as compared to inhibition caused by antibodies against a hybrid protein of AMA1 and MSP1 (dark bars). Bars represent the mean of three experiments; error bars indicate the standard deviation.

Invasion inhibition assays in which late stage parasites were grown in the presence of α-Pf332-DBL/nDBL-antibodies visualized the influence of Pf332 on parasite invasion. For the three different parasite clones/strains FCR3S1.2, 3D7AH1 and 7G8, the antibodies showed an inhibition effect on invasion, with reduction to around 60% observed at concentrations of 1 mg/ml (Fig. 6), this effect was declining with decreasing concentrations of the α-Pf332-DBL/nDBL-antibodies. Antibodies against MSP-1 and AMA-1, known antigens involved in RBC invasion [6], [7] revealed a similar inhibition effect when used in the same invasion inhibition assays. Although the influence of the α-Pf332DBL/nDBL-antibodies was slightly lower, these antibodies gave a similar pattern compared to the α-AMA-1/MSP-1 antibodies (Fig. 6), indicating a role of Pf332 during the reinvasion process of the merozoites.

## Discussion

Successful attachment of the *P. falciparum* merozoite to the RBC surface is the first important step in the process of invasion and intracellular proliferation. Numerous *in vitro* studies have concluded that the cascade of the rapid invasion process, taking less than 20 seconds [26], is complex and involves a large number of different molecules. Proteins such as the MSPs, AMA-1, EBL- and Rh-family members located in the micronemes, the rhoptries or on the merozoite surface have been shown to play a role in attachment or invasion of the merozoite. However, many aspects of this process remain to be elucidated. The molecule initiating the contact between the merozoite and the RBC is not identified, and the exact location of the invasion process within the human body is still unknown. Furthermore, it is at present unclear as to which molecular events underlie the loss of infectivity, which is observed in merozoites isolated from *in vitro* grown *P. falciparum* parasites.

To shed additional light on the complex process of invasion we have used the genome sequence of *P. falciparum* to identify proteins potentially involved in invasion and discovered the ORF PF11_0506 that possesses a RBC-binding domain. Detailed analysis of this gene fragment and the encoded protein has led to several discoveries.

We have here revised the gene structure of Pf332. RT-PCR and Northern blot clearly showed that the two predicted ORF PF11_0506 and PF11_0507 are two exons of a single gene. This conclusion was confirmed by Western blot analysis using three different α-Pf332 antibodies to various regions of the polypeptide. Exon II of Pf332 was initially identified from a gDNA expression library containing only the region of repetitive sequence without additional 5′-RACE sequencing to identify the 5′-end of the transcript [15], [27]. The existence of the additional upstream exon in the gene of Pf332 has therefore previously been overlooked and exon I (PF11_0506) was as a consequence annotated in the *P. falciparum* genome as a separate gene encoding a hypothetical protein. Our revision of the gene structure of Pf332 has paved the way to dissect the biological function of Pf332, one of the largest proteins found in *P. falciparum* parasites.

The first exon of Pf332 encodes a conserved erythrocyte-binding domain homologous, but with a distinct sequence, to the DBL-domains of the EBL-family. This sequence is, in contrast to other erythrocyte-binding proteins, conserved among different *P. falciparum* strains and clones. Proteins expressed by the *Plasmodium* parasite often show sequence alterations in regions that are crucial for immunity or undergo antigenic variation, in particular surface related molecules, due to the constant pressure from the host immune system. This also includes members of the EBL-family, as for example the EBA-175, that are conserved only in function but show variation within their sequence [28]. However, the extracellular region of Pf332 displays a conserved sequence, which might ensure the parasite's ability to adhere to RBC independent of the genetic background of the host. The capacity to bind uninfected RBC to the schizont surface might act as a virulence factor for the parasite, and greatly increase the invasion efficiency. Due to the conservation of sequence in the DBL-domain this virulence factor might be primarily of importance in non-immune individuals without prior exposure to the parasite, rapid acquisition of antibodies against this invariable domain in semi-immune individuals could contribute substantially to protection against high multiplication rates of the parasite and therewith against severe disease.

A common feature of *P. falciparum* is that this parasite is able to invade any human RBC, although the efficiency generally depends on the carbohydrate moieties of the glycophorin on the RBC surface. It can be hypothesized that the initial contact between schizont and the RBC is the interaction of a conserved receptor-ligand pair, a function that could be fulfilled by the DBL-domain of Pf332 and an unknown receptor on the RBC surface, while the invasion afterwards is dependant on molecules such as MSP-1 and AMA-1. Numerous laboratory and patient isolates are frequently found to form rosettes during late trophozoite and schizont stage. This rosette formation is irrespective of the “classical rosetting” mediated by PfEMP1 taking place earlier in the parasites lifecycle. Merozoites of these parasites invade more efficiently uninfected RBC involved in these rosettes than into unattached RBC (data not shown). The association of the DBL-domain of Pf332 with the iRBC surface correlates with late stage rosetting, indicating that Pf332 might facilitate invasion for released merozoites through providing close proximity of new host cells. Even though schizonts are often located in capillaries, where the speed of blood flow is reduced due to sequestered and agglutinated iRBC, the newly released merozoites would additionally benefit from a reduced distance towards new host cells. Pf332 might in addition support the invasion process of merozoites after rupture of the schizont. Accumulating amounts of Pf332 during schizont stage might loosely attach to the merozoites surface assisting the parasite to more easily come in contact with and to invade a new host cell after release from the schizont (Fig. S4).

It has previously been suggested that the EB200 region of Pf332 with its degenerative repeats might function as an immunological smokescreen distracting antibodies away from important epitopes of the molecule [29]. In contrast to the DBL-domain, exon II displays sequence polymorphism among different isolates possibly attracting the host immune response and shielding the functional N-terminal region of the molecule. Subsequently, the DBL-domain of Pf332, although exposed in some way to the immune system, is able to retain a conserved sequence mediating the important first step of the process of RBC invasion. Still, the lack of variation of the DBL-domain suggests that it is not overtly exposed to the immunesystem as PfEMP1 or the RIFINs, but concealed prior to exerting it function.

Quantitative differences in expression of Pf332 have been reported from different *P. falciparum* strains in earlier studies [30]. Microarray data presented in the PlasmoDB (http://www.plasmodb.org) show Pf332 to be transcribed in 3D7, Dd2 and HB3 parasites with a higher transcription level in HB3 iRBC than in the other strains. Interestingly, the chromosomal fragment containing the gene of Pf332 is duplicated in the HB3 strain [31]. Here we confirm the transcription and expression of Pf332 by both real-time quantitative PCR and semi-quantitative Western blot analysis. In our quantitative PCR assays, the transcription level of the gene was related to the transcription of *seryl-tRNA synthetase*, a house-keeping gene which is constantly active during the whole erythrocytic cycle of the parasite making it a suitable control for time course studies of gene expression. RNA was collected every 4 hours from 8 h p.i. onwards and transcription of the Pf332 gene was found to start at 16 h p.i. as described before [18], [32]. While the transcription pattern was similar in all parasite clones/strains, the transcription level observed in FCR3S1.2 iRBC was more than twice as high as in the other two strains (Fig. 3A), resulting in a significantly higher expression of Pf332 as visualized in Western blot assays (Fig. 3C) indicating a direct relation between Pf332 transcription and expression. Interestingly, among the parasite clones/strains investigated, the FCR3S1.2 clone has the shortest replication cycle (44–46 h instead of 48 h), and in addition an approximately doubled multiplication rate compared to 3D7AH1 iRBC (data not shown) suggesting a potential benefit from increased amounts of Pf332.

To date, the only parasite-derived protein on the iRBC surface that is well characterized is the molecule PfEMP1. However, PfEMP1 is mainly expressed in the earlier trophozoite stage and its foremost function is to mediate adhesion of the iRBC to the vascular endothelium. Pf332 is in contrast expressed at late trophozoite and schizont stage and proposed to be involved in binding of uninfected RBC. Immunofluorescence assays performed in this study and earlier [18] show that Pf332 first accumulates in the parasitophorous vacuole and is thereafter, transported into the erythrocyte cytoplasm where it meets with PfEMP1 and the RIFINs on their way towards the surface of the iRBC. The Pf332 molecule contains four variant antigen-specific translocation signal sequences (PEXEL motifs) [22], [23] indicating that this molecule is transported to the RBC surface. While Pf332 and PfEMP1 transiently co-localize in the late trophozoite stage, their distribution pattern within the iRBC again differs during the schizont stage reflecting functional differences of the two molecules. Pf332 is presented later on the surface of the iRBC than PfEMP1 and may participate in the sequestration process of the iRBC in the microvasculature as previously suggested [16]; however its main function is to facilitate merozoite invasion. This is further supported by our data obtained by quantitative PCR, which illustrate that the Pf332 gene is activated at a later time point during the erythrocytic life cycle than the *var*-genes. Even though the DBL-domain of Pf332 is homologous to RBC invasion-related proteins such as EBA-175 it clearly differs from these microneme- and rhoptry-related molecules regarding the time of transcription initiation (starts at around 38 h p.i. for these proteins, PlasmoDB). Hence, Pf332 displays a distinct expression profile, clearly distinguishing it from other *P. falciparum* surface molecules pointing at a unique function of this molecule in the parasite life cycle. Pf332 presents a novel type of invasion-related molecule facilitating and supporting the invasion of future merozoites, clearly different from other cytoadhesion-related proteins such as PfEMP1 or merozoite invasion ligands (e.g. MSPs).

Despite the major interest in Pf332, its characterization has been hampered by the fact that many of the α-Pf332 antibodies are cross-reactive with other molecules, e.g. the Pf155/RESA also containing regular repeats of glutamic acid [33]. Western blot analysis of our antibodies raised against the DBL/nDBL-region of the newly discovered exon I of Pf332 has revealed that these antibodies are specifically reacting with the Pf332. They do not recognize or cross-react with any other parasite antigens neither in Western blot nor immunoflurescence assays carried out on merozoites (Fig 3C, 4E and data not shown) supporting the conclusion that the reduction of invasion efficiency of the α-Pf332-DBL/nDBL antibody is specific. We could show that both recombinant protein and antibodies to the DBL-domain of Pf332 reduce parasite invasion, while antibodies towards the PfEMP1 (α-DBL1-domain) did not (Fig. 6 and data not shown). Comparing the effect of our α-Pf332 antibodies with antibodies known to target the process of invasion [6], [7], a similar inhibitory effect is observed, indicating that Pf332 might harbour a function during the merozoite invasion process.

In conclusion, we have identified a molecule with a conserved RBC-binding DBL-domain that plays an important role in invasion of the *P. falciparum* parasite. The protein is apparently essential for the efficient proliferation of all *P. falciparum* parasites. This discovery can have important implications in malaria vaccine development as well as in designing new drugs to block parasite invasion.

## Materials and Methods

### Bioinformatic analysis of the upstream sequence of the Pf332 gene in the 3D7 genome

Analysis of the 3D7 parasite genome for sequences containing RBC-binding motifs led to the identification of a hypothetical gene (PF11_0506) located 281 bp upstream of the gene Pf332, which potentially encodes a RBC-binding domain. PF11_0506 has one ORF with a size of 1710 bp. The putative sequence of this polypeptide was aligned with sequences of several EBL-family proteins. The secondary structure and the cellular locations were analysed with the Macvector program (GCG, UK).

### Gene transcription and analysis

From total RNA purified with TRIZol®[34] first strand cDNA was synthesized (GeneAmp RNA PCR Kit, Applied Biosystems, USA) and further amplified with the primers UP3 (TAG TAC CAG GAG TAT TAA CA) and UP4 (TTC CGT GTA TCT TCT TCT TC) (Fig. 1). Northernblot was carried out with two anti-sense digitonin-labelled RNA probes as described [4]. The RNA probes were generated from two plasmids; plasmid 1 containing a sequence amplified with primers UP1 (ATG TCT AAT ATA AAT AAC AAA GAC TC) and UP2 (AGA ATT CAT CAC AAC TCT CAT); plasmid 2 with a fragment amplified from cDNA with primer UP3 and UP4 (Fig. 1).

### Gene amplification and sequence analysis

To investigate the polymorphism of exon I, gDNA was purified (from FCR3S1.2, 3D7AH1, NF54CSA, FCR3CSA, TM284, R29, Dd2, K1, F32, FCB-3 and the Ugandan isolate UAS22, standard methods) and PCR-amplification was carried out with primers UP1 (ATG TCT AAT ATA AAT AAC AAA GAC TC) and UP5 (TAT TAC CTT ATA TAC CAA GAC C) (Fig. 1). Products were cloned into the pCRII vector (Invitrogen USA) for sequencing.

### Real-time quantitative PCR analysis of Pf332 transcription

RNA was harvested at 4 h p.i. intervals (RNeasy® Mini Kit, Qiagen, Valencia, CA) and reverse transcriped (SuperScript III RNase H reverse transcriptase, Invitrogen). For real-time quantitative PCR reactions to monitor the relative expression of Pf332 to the endogenous control *seryl-tRNA synthetase* we employed an MGB-probe approach. Primers and probes were for Pf332: AAG AAG ATG TGG GAT GTG TTC CA, CAT TTT CAT TAT CCA ACC TTT CCA T and 6-FAM-CTA GGAG ACA GAA TTT GA-MGB and for *seryl-tRNA synthetase*: TAT CAT CTC AAC AGG TAT CTA CAT CTC CTA, TTT GAG AGT TAC ATG TGG TAT CAT CTT TT and 6-FAM-AAA GAT ATC ATC ACA GGC AGA T-MGB. Amplification reactions were done in quadruplicates in MicroAmp 96 well plates in 20 µl, containing TaqMan buffer with UNG (Applied Biosystems), 900 nM of each forward and reverse primer, 200 nM of each probe and 2 ng of template. 45 cycles (95°C for 15 sec and 60°C for 1 min) were performed in an ABI sequence detector 7500 (Applied Biosystems). The detection threshold was set above the mean baseline value for the first 6-15 cycles. Amplification efficiencies were verified by performing amplifications using different concentrations of cDNA for the target (Pf332) and reference (*seryl-tRNA synthetase*) and were sufficiently close to obviate the need for a correction factor. Results were analyzed by the Relative Standard Curve Method where a normalized target value was achieved by dividing the *x̅*_target_ with the *x̅*_reference_ for each clone/strain and time point. The standard deviation of the quotient was calculated according to the User Bulletin 2 (Applied Biosystems, http://www.appliedbiosystems.com). Results were visualized as log_2_ transformed values plotted using SigmaPlot 9.0 (Systat Software Inc.). Statistically significant strain differences in Pf332 transcription were determined for each time point performing group comparisons with One Way ANOVA and the Tukey test.

### Generation of recombinant Pf332-DBL- and nDBL-fusionproteins and binding to human RBC

GST- or his-fusion proteins were generated by cloning the ORF of the DBL- and nDBL-domain into the pGEX-4T-1- (Amersham Biosciences, Sweden, primer: Pf332-DBL: forward: *GGA TCC* AGC AAC ATC AAC AAC AAG GA and reverse: *CTC GAG* GTA CTT CTT CTC GAA CAC C; Pf332-nDBL: forward: *GGA TCC* GCT AAT GAT AAT AAA TCA AAA CA and reverse: *CTC GAG* TAT TTC CTG CAT TCA TTC C) or pQE60-vector (Qiagen) (primer F-DBL: *GCA TGC* GAA GCA ACA TCA ACA ACA AGG, R-DBL: *GGA TCC* GTA CTT CTT CTC GAA CAC C) and recombinant proteins were expressed as described [35].

Recombinant protein (200 pmol) was incubated with 5 µl of washed RBC in RPMI for 2.5 h at 4°C, washed and bound protein was visualized by Westernblot (α-GST mAb, dilution 1∶5000, Sigma; α-mouse mAb-ALP, dilution 1∶10000, Sigma). In addition, RBCs were treated with neuraminidase (0.2 U in PBS for 1 h at 37°C), heparitinase (0.5 U in 50 mM Tris for 1 h at 37°C) or trypsin (100 µg/ml in PBS for 1 h at 37°C) in a 20 µl scale before the binding assays.

### Surface-expression of the Pf332-DBL- and Pf332-nDBL-domain on CHO-cells and binding to human RBC

The Pf332-DBL- (optimized for expression in mammalian cells, GeneArt, Germany) and Pf332-nDBL-domain were cloned into the pDisplay vector (Invitrogen). Transfection of CHO-cells was performed with the FuGENE 6 transfection reagent (Roche Applied Science, Switzerland) and expression of the domains on the cell surface was confirmed by immunofluorescence 48 h later (α-HA-antibody, Invitrogen; Alexa-labelled-α-mouse-antibody, Molecular probe, USA).

For detection of RBC-binding transfected CHO-cells were detached, and stained with PKH67 (green); human RBC were in parallel stained with PKH26 (red). CHO-cells were incubated with RBC at a ratio of 1∶5 for 1 h at RT and the rosette formation was afterwards analysed by fluorescence microscopy.

### Generation of specific antibodies against the region encoded by exon I of Pf332

The region encoded by exon I of Pf332 (aa 1–533) was cloned into the Semliki forest virus vector SFV3.spider (primer: 332-F-SFV (*CCC GGG* ATG TCT AAT ATA AAT AAC AAA GAC and 332-RII-SFV (*CCC GGG* ATA ATT TCC TGC ATT CAT TCC ATC) and virus particles generated as described before [36]. Rabbits (New Zeeland white) or rats were immunized (three times 5×10^8^ particles/animal, once 500 µg protein/animal, respectively four times 100 µg protein/animal).

### Immunodetection of Pf332 in Westernblot and immunofluorescence on fixed and live iRBC

Trophozoite iRBC were purified [37], [38], lysed in SDS-loading buffer, analyzed by 6% SDS-PAGE (2×10^6^ cells/lane) and subjected to various α-Pf332 sera. Visualization was performed with an α-rabbit Ig- (1∶5000, Amersham) or an α-human Ig-HRP-conjugate (1∶1500, Amersham).

For immunofluorescence assays, monolayers of iRBC were prepared as described before [18]. Monolayers were incubated 30 min with the α-Pf332-DBL/nDBL antibody (1∶800) or pre-immune serum (1∶800), washed three times in PBS, and incubated 30 min with a secondary antibody (α-rabbit-Alexa, Molecular Probes, 1∶100).

For live IFA-assays, monolayers were prepared on adhesive slides (Marienfeld Glassware) according to the manufacturer's protocol. Negatively charged cells are bound to these slides based on electrostatic adhesion without additional fixation steps allowing the investigation of live late-stage iRBC. The cells were incubated with the α-Pf332-DBL antibody (1∶50), pre-immune serum (1∶50), α-PfEMP2 (1∶50) or serum to an unrelated control protein (1∶50), washed three times in PBS, and incubated with a secondary antibody (α-rat-Alexa, Molecular Probes, dilution 1∶300) and counterstained with Ethidium-bromide. All incubations were carried out at room temperature in a humid chamber. Slides were analyzed with a 100×oil immersion lens in a Nikon Optiphot 2 UV microscope.

### Invasion inhibition assay

IRBC of various parasite clones/strains at trophozoite stage were adjusted to a parasitemia of 1% and a hematocrit of 2.5%, mixed with the purified IgG fraction of an α-Pf332-DBL/nDBL-, α-GST- or α- AMA1/MSP1-serum [39] in final concentrations of 1, 0.5 and 0.25 mg/ml (in duplicates) and cultivated in 96-well plates until reinvasion of merozoites was completed. IRBC were stained with Acridine Orange and counted (50,000 events) by flow cytometry. Experiments were repeated three times and the effect was determined by relating the observed parasitemia in the presence of α-Pf332-DBL/nDBL- or α-AMA1/MSP1-antibodies to α-GST antibodies (negative control).

## Supporting Information

Figure S1 The sequence of the exon I of Pf332 is conserved among various parasite clones/strains. PCR amplification of the exon I region of the Pf332 gene from gDNA of various parasites showed a single product of 1710 bp (primer UP1 and UP5; compare Fig. 1A) from 10 different P. falciparum clones/strains and one Ugandan isolate. Enzymatic treatment of the amplified fragments with EcoR I illustrated that there was only one sequence in each amplicon. All amplified products were cloned and sequenced and only 4 mutations were identified among the 11 sequences (compare table 1).
(0.24 MB TIF)

Figure S2 Alignment of the amino acid sequence of the EBL-family members. The sequence of Pf332 encoded by exon I shares numerous amino acid residues (highlighted with stars) that are conserved among the erythrocyte-binding proteins of different Plasmodium-species such as the sequences of the DBL-domain of the P. vivax Duffy binding protein (DBP), P. knowlesi DBP, P. cynomolgi DBP, BAEBL/EBA-140, JESEBL/EBA-181 and EBA-175 of P. falciparum
(0.31 MB TIF)

Figure S3 Immunoblot analysis of the molecule Pf332. Antibodies towards the repetitive region EB200 and the monoclonal antibody m33G2 have previously been used to characterize the molecule Pf332. These antibodies react with the same high molecular weight polypeptide as were recognized by antibodies raised towards the DBL/nDBL-region of Pf332. The same recognition pattern could be observed in all parasite strains/clones (3D7AH1, 7G8, FCR3S1.2) investigated. The band corresponding to Pf332 is marked with an asterix.
(0.88 MB TIF)

Figure S4 Schematic model of the role of the DBL-domain of Pf332 during merozoite invasion. A: The DBL-domain of Pf332 is exposed on the late stage iRBC surface and mediates binding to uninfected RBC causing a “late resetting” phenotype. This process provides close proximity of new host cells and facilitates invasion of the released merozoites into new RBC. B: The molecule Pf332 is surface associated. It accumulates during schizont stage and attaches loosely to the merozoites surface assisting the parasite to more easily come in contact with and to invade a new host cell after release from the schizont
(0.32 MB TIF)
