# Supplementary figures and images for: A Novel DBL-Domain of the P. falciparum 332 Molecule Possibly Involved in Erythrocyte Adhesion
Source: PLoS One. 2007 May 30;2(5):e477. doi: 10.1371/journal.pone.0000477 (PMC1868959; doi:10.1371/journal.pone.0000477)

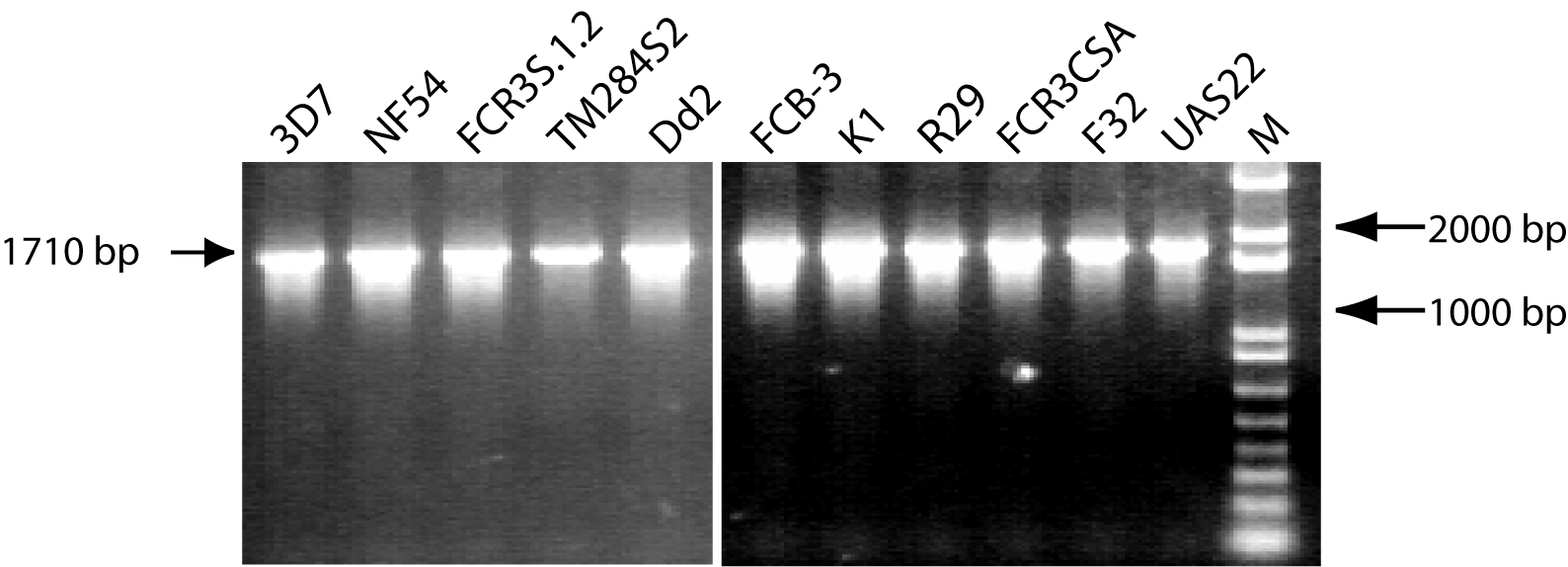

Supplement: Figure S1 — The sequence of the exon I of Pf332 is conserved among various parasite clones/strains. PCR amplification of the exon I region of the Pf332 gene from gDNA of various parasites showed a single product of 1710 bp (primer UP1 and UP5; compare Fig. 1A) from 10 different P. falciparum clones/strains and one Ugandan isolate. Enzymatic treatment of the amplified fragments with EcoR I illustrated that there was only one sequence in each amplicon. All amplified products were cloned and sequenced and only 4 mutations were identified among the 11 sequences (compare table 1). (0.24 MB TIF) [file pone.0000477.s001.tif]

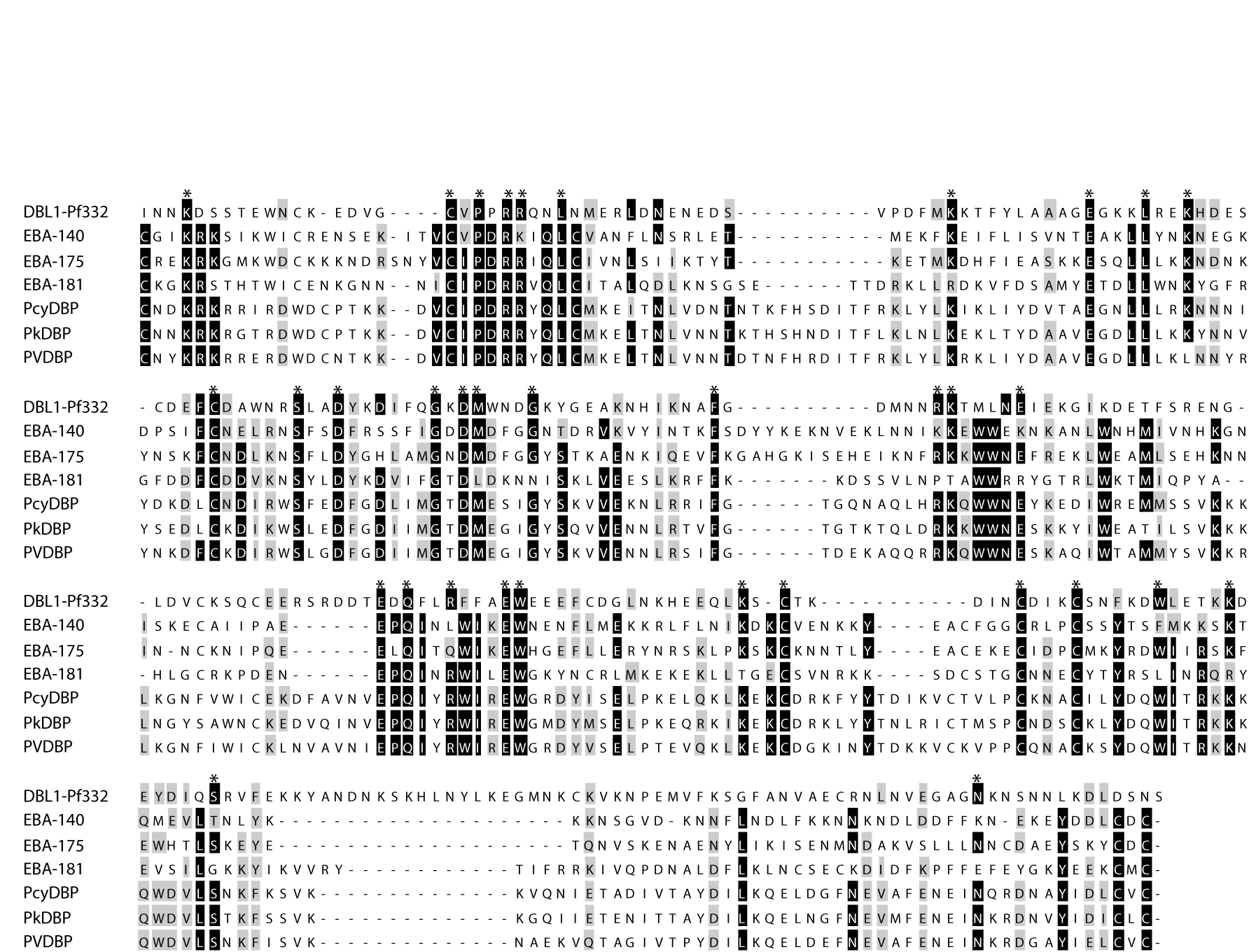

Supplement: Figure S2 — Alignment of the amino acid sequence of the EBL-family members. The sequence of Pf332 encoded by exon I shares numerous amino acid residues (highlighted with stars) that are conserved among the erythrocyte-binding proteins of different Plasmodium-species such as the sequences of the DBL-domain of the P. vivax Duffy binding protein (DBP), P. knowlesi DBP, P. cynomolgi DBP, BAEBL/EBA-140, JESEBL/EBA-181 and EBA-175 of P. falciparum (0.31 MB TIF) [file pone.0000477.s002.tif]

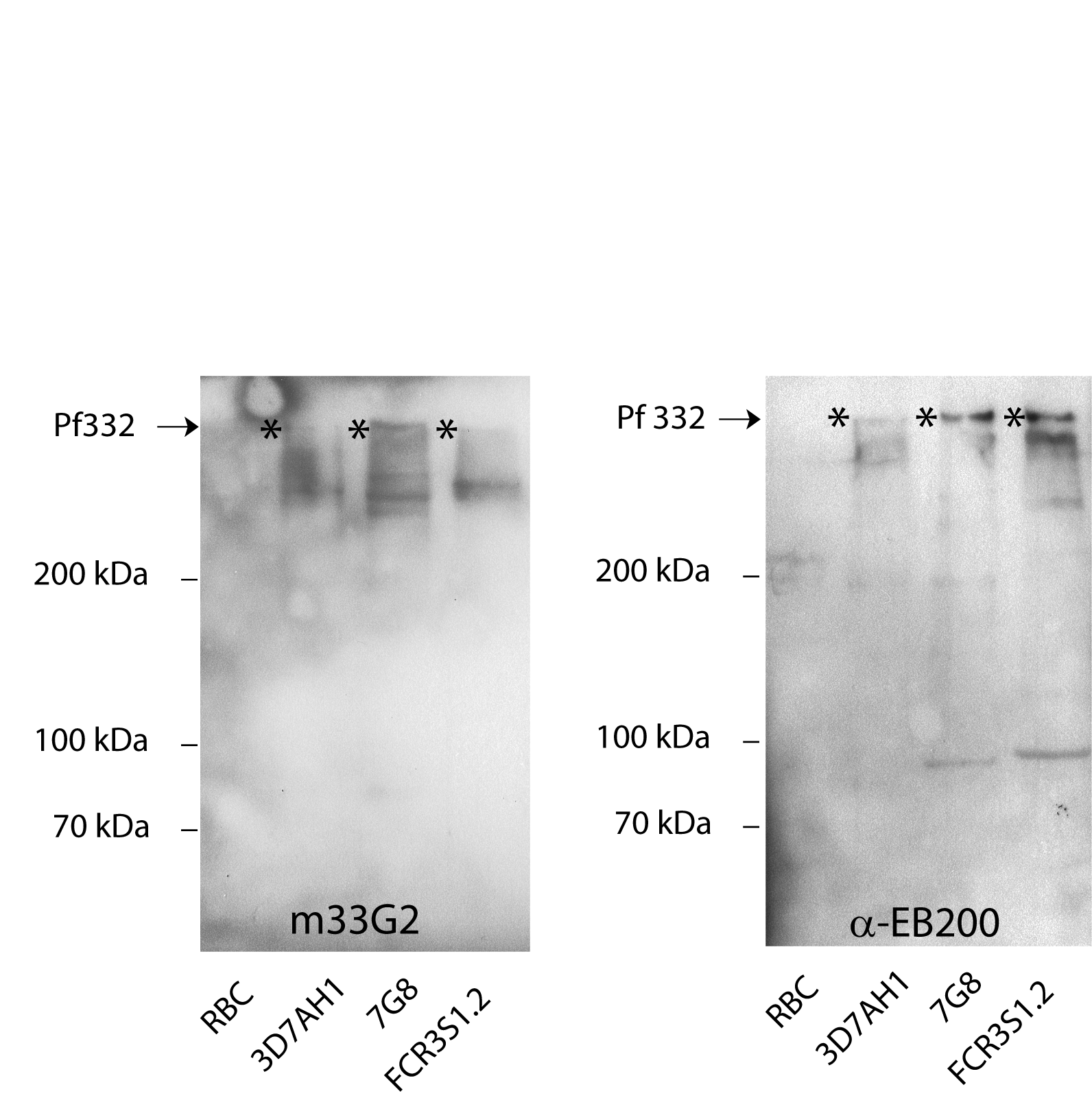

Supplement: Figure S3 — Immunoblot analysis of the molecule Pf332. Antibodies towards the repetitive region EB200 and the monoclonal antibody m33G2 have previously been used to characterize the molecule Pf332. These antibodies react with the same high molecular weight polypeptide as were recognized by antibodies raised towards the DBL/nDBL-region of Pf332. The same recognition pattern could be observed in all parasite strains/clones (3D7AH1, 7G8, FCR3S1.2) investigated. The band corresponding to Pf332 is marked with an asterix. (0.88 MB TIF) [file pone.0000477.s003.tif]

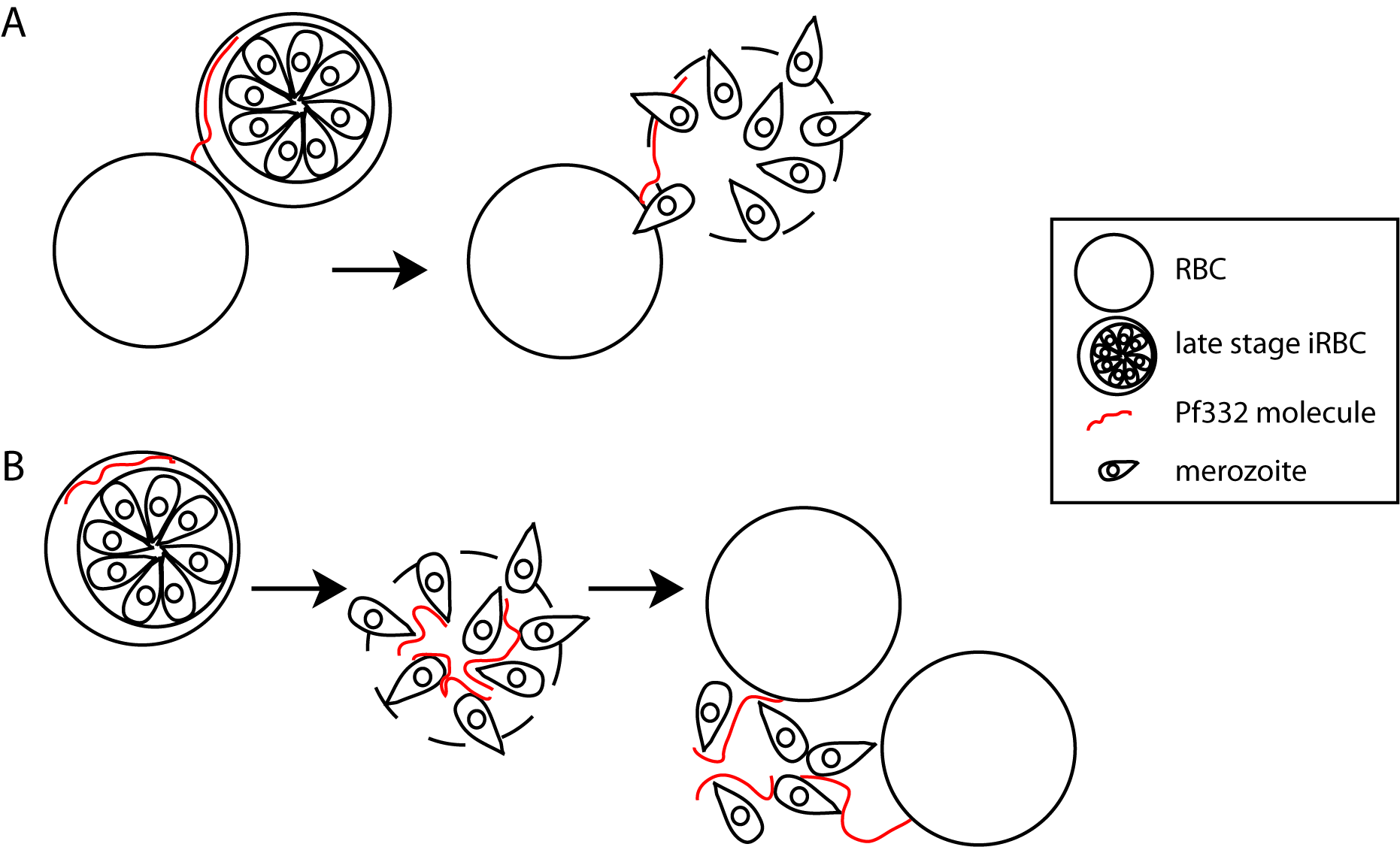

Supplement: Figure S4 — Schematic model of the role of the DBL-domain of Pf332 during merozoite invasion. A: The DBL-domain of Pf332 is exposed on the late stage iRBC surface and mediates binding to uninfected RBC causing a “late resetting” phenotype. This process provides close proximity of new host cells and facilitates invasion of the released merozoites into new RBC. B: The molecule Pf332 is surface associated. It accumulates during schizont stage and attaches loosely to the merozoites surface assisting the parasite to more easily come in contact with and to invade a new host cell after release from the schizont (0.32 MB TIF) [file pone.0000477.s004.tif]
